# Production parameters and pig production cost: temporal evolution 2010–2014

**DOI:** 10.1186/s40813-016-0027-0

**Published:** 2016-04-11

**Authors:** J. Rocadembosch, J. Amador, J. Bernaus, J. Font, L. J. Fraile

**Affiliations:** 1 SIP consultors SL, Prats del Lluçanes, Spain; 2 Departamento de Medicina y Zootecnia de Cerdos, FMVZ, Universidad Nacional Autónoma de, México, Mexico; 3 Departament de Producció Animal, ETSEA, University de Lleida, Lleida, Spain; 4 AgrotecnioCenter, Lleida, Spain

**Keywords:** Parameter production, Cost, Pig, Company size, Evolution

## Abstract

**Background:**

The assessment of the cost of production and the relative weight of the different production parameters is very important in pig farming. The goals of the present work were 1) to describe reliable reference values for production parameters and pig production cost from 2010 to 2014, 2) to describe their temporal evolution and 3) to determine the influence of the pig company size on them. Between 61 and 107 pig production companies from Spain were included in this study from 2010 to 2014. These companies sent data on feed consumption, number of pig produced, expenses and census every month. Sip consultors SL standardized collected data and calculate cost and production parameters to obtain values comparables between the different pig production companies. The collected data each month were merged to obtain a yearly average value taking into account the pig production flow each month. A suitable statistical analysis was carried out to tackle the goals.

**Results:**

The production performance has been continuously improving in the piglet production and fattening phase from 2010 to 2014. Thus, the number of piglets by sow and year will increase 0.5 pigs by year and the total feed conversion rate will decrease approximately 0.03 kg feed/kg gain by year in the future if the same tendency continues. However, feed price has been steadily increasing from 2010 to 2012 and decreasing afterwards and the total cost per kilogram produced has followed a similar pattern. This result highlights the relevance of the feed price in the final cost in spite of continuous improvement in production performance across years. Finally, pig company size affected most of the production parameters studied. Thus, the best technical parameters were obtained for companies with less than 5000 sows. However, the opposite tendency is observed for feed price where the highest value was observed for the smallest companies.

**Conclusions:**

Pig production parameters have generally improved in the last five years but this improvement did not directly imply a reduction in pig production cost due to the high feed prices during the period 2010–2013.

**Electronic supplementary material:**

The online version of this article (doi:10.1186/s40813-016-0027-0) contains supplementary material, which is available to authorized users.

## Background

The pork industry is facing lower profit margins per pig, or negative profits with prices lower than marginal production costs, from time to time. Therefore, economically rational decisions should be based on an assessment of private costs and benefits at the producer level. Moreover, the relative weight of the different production parameters must be deciphered in the final cost of pig production to decrease the cost as much as possible and increase competitiveness in the future. Spain is the second and fourth pig producer in Europe and the world, respectively and it becomes one of the main global players in the pig market due to its great potential for exportation to Europe and Asian countries [1]. Thus, evolution of production parameters and pig production cost in Spain is interesting not only at national but also at international level.

Performance indicators routinely used to measure pigs’ productivity include average daily gain (ADG) and feed conversion ratio (FCR) during the nursery and fattening phase [2]. In addition, increased mortality in growing pigs is also related to decreased profitability in swine operations [3]. Thus, those indicators can be used to quantify the impact of any disease during the rearing period [4]. Reliable performance indicators will help to quantify the impact on any disease on the swine industry and to estimate the cost-effectiveness of alternative prevention and control measures focus on prioritizing the use of resources [5–7]. There is a scarcity of information about pig production parameters and cost that could be used as a reference probably due to the complexity inherent in gathering information coming from different pig companies which are competitors in the same market. The goals of the present work were 1) to describe reliable reference values for production parameters and pig production cost from 2010 to 2014, 2) to describe its temporal evolution and 3) to determine the influence of the pig company size on them.

## Results

### Piglet production phase

**Fig. 1:**
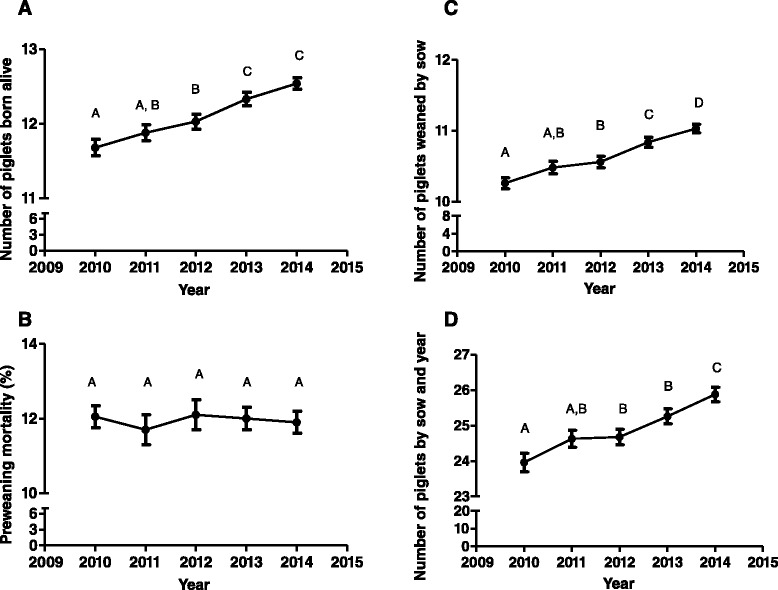
Temporal evolution of number of piglets born alive (**a**), preweaning mortality (**b**), number of piglets weaned by sow (**c**) and number of piglets produced by sow and year (**d**) during piglet production phase from 2010 to 2014 in Spain. Values with different superscripts differ significantly between years at *P* < 0.05

**Fig. 2:**
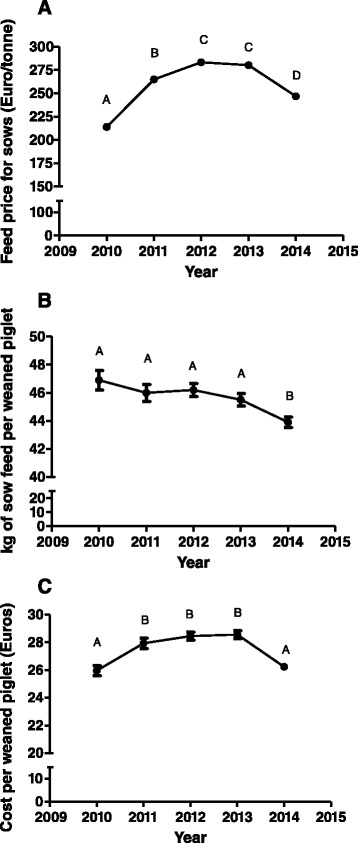
Temporal evolution of sow feed cost (**a**), kilograms of sow feed per weaned piglet (**b**) and cost of piglet production (**c**) during piglet production phase from 2010 to 2014 in Spain. Values with different superscripts differ significantly between years at *P* < 0.05

The number of piglets per sow and year has been increasing every year due to a parallel increase in the number of piglets born and weaned by sow during each production cycle. Curiously, this increase in prolificacy has not implied an increase in the preweaning mortality across years (Fig. 1). However, sow feed price and the cost of production of a weaned piglet have been steadily increasing from 2010 to 2012 and decreasing afterwards (Fig. 2). In any case, sow feed price in 2014 was significantly higher than in 2010. On the other hand, kilograms of sow feed per weaned piglet have been steadily decreasing in the last five years. Pig company size was significantly affecting most of the parameters studied with the exception of number of cycles by sow and year (Table 1). Curiously, in general terms, the best technical parameters were obtained for companies with less than 5000 sows (Table 1). However, the bigger the pig size company is, the lower the sow feed price is. Nevertheless, the cost per weaned piglet in the smallest companies (<5000 sows) is significantly lower than in the biggest ones (>5000 sows). Finally, a complete descriptive statistics is provided by year in this phase (Additional file 1: Table S1 and S2).

**Table 1:** Average (+SEM) shown for production parameters and pig production cost by pig company size from 2010 to 2014 in Spain

	Pig company size (number of sows)			
Parameter	<1000	1000–5000	5000–10.000	>10.000
NBA	12.22 ± 0.08 ^a^	12.24 ± 0.08 ^a^	12.04 ± 0.13 ^a,b^	11.92 ± 0.12^b^
PM1 (%)	12.27 ± 0.32 ^a^	11.78 ± 0.23 ^ab^	12.39 ± 0.45 ^ab^	11.16 ± 0.30 ^b^
NW	10.71 ± 0.06 ^a,b^	10.79 ± 0.07 ^b^	10.54 ± 0.10 ^a^	10.58 ± 0.07 ^a^
NPWY	24.97 ± 0.17 ^a,b^	25.30 ± 0.19 ^a^	24.59 ± 0.27 ^b^	24.89 ± 0.22 ^a,b^
NCS	2.33 ± 0.01 ^a^	2.34 ± 0.01 ^a^	2.33 ± 0.01 ^a^	2.34 ± 0.01 ^a^
FP1	261.86 ± 2.42 ^a^	260.11 ± 2.32 ^a,b^	254.50 ± 4.04 ^a,b^	251.87 ± 3.62 ^b^
KFWP	44.96 ± 0.46 ^a^	45.19 ± 0.33 ^a^	46.77 ± 0.52 ^b^	46.43 ± 0.52 ^b^
TSF	1116.16 ± 7.77 ^a^	1136.84 ± 6.13^a,b^	1144.50 ± 7.54 ^b^	1150.94 ± 7.91 ^b^
CWP1	26.95 ± 0.25 ^a^	27.19 ± 0.27 ^a^	28.59 ± 0.30 ^b^	27.90 ± 0.29 ^b^
ADG2	299.14 ± 3.63 ^a^	289.89 ± 3.27 ^a,b^	289.45 ± 5.27 ^a,b^	278.11 ± 5.36 ^b^
FCR2	1.64 ± 0.01 ^a^	1.67 ± 0.01 ^a^	1.66 ± 0.01 ^a^	1.72 ± 0.01 ^b^
NM2 (%)	3.18 ± 0.14 ^a^	3.16 ± 0.13 ^a^	3.15 ± 0.16 ^a^	3.34 ± 0.16 ^a^
FP2	476.44 ± 4.11 ^a^	469.04 ± 4.09 ^a,b^	438.80 ± 5.30 ^c^	420.64 ± 5.35 ^d^
CNP2	41.76 ± 0.37^a^	42.07 ± 0.34^a^	43.33 ± 0.40^b^	42.27 ± 0.30^a,b^
DVCNP2	3.00 ± 0.09^a^	3.48 ± 0.07^b^	3.62 ± 0.12^b,c^	3.22 ± 0.07^d^
ADG3	659.50 ± 4.70 ^a^	656.81 ± 6.24 ^a^	668.22 + 16.4 ^a^	625.24 ± 7.88 ^b^
FCR3	2.64 ± 0.01 ^a^	2.65 ± 0.01 ^a^	2.66 ± 0.02 ^a^	2.65 ± 0.02 ^a^
FM3 (%)	3.75 ± 0.20 ^a^	3.72 ± 0.14 ^a^	4.06 ± 0.16 ^a^	3.94 ± 0.16 ^a^
FP3	287.68 ± 2.79 ^a^	287.20 ± 2.64 ^a^	283.99 ± 4.17^,ba^	276.49 ± 3.95 ^b^
TCP3	123.52 ± 0.96 ^a^	124.81 ± 0.91 ^a,b^	126.76 ± 1.26 ^b^	122.74 ± 1.17 ^a^
DVCFP3	1.22 ± 0.09^a^	1.72 ± 0.08^b^	1.97 ± 0.10^c^	1.67 ± 0.08^b,d^
FCRT	2.80 ± 0.01 ^a^	2.82 ± 0.01 ^a^	2.83 ± 0.02 ^a^	2.84 ± 0.02 ^a^
TCK	1.15 ± 0.01 ^a,b^	1.16 ± 0.01 ^a,b^	1.18 ± 0.01 ^b^	1.14 ± 0.01 ^a^
TFC	89.06 ± 0.91 ^a^	89.52 ± 0.77 ^a^	88.07 ± 1.17 ^a,b^	86.11 ± 1.16 ^b^
DVCT	4.39 ± 0.18 ^a^	5.30 ± 0.14 ^b^	5.52 ± 0.19 ^b,c^	5.02 ± 0.13 ^d^
TFIXC	27.98 ± 0.44 ^a^	27.86 ± 0.27 ^a,b^	30.47 ± 0.48 ^c^	29.25 ± 0.25 ^d^
TREPC	2.51 ± 0.12 ^a^	2.53 ± 0.09 ^a^	3.06 ± 0.11 ^b^	2.72 ± 0.12 ^b^

Abbreviations are defined in Table 3

Values with different superscripts differ significantly between pig company size at *P* < 0.05

### Nursery phase

**Fig. 3:**
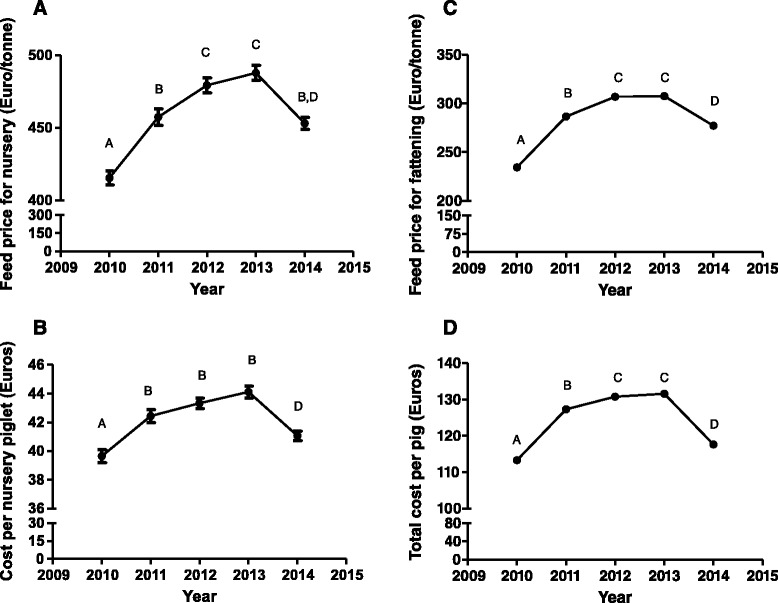
Temporal evolutions of feed cost and cost of production during nursery (**a** and **b**) and fattening phase (**c** and **d**) from 2010 to 2014 in Spain. Values with different superscripts differ significantly between years at *P* < 0.05

Average daily gain, feed conversion and mortality rate have not changed during the nursery phase from 2010 to 2014 (Additional file 1: Figure S1). However, nursery feed price has been increasing steeply from 2010 to 2013 and decreasing subsequently (Fig. 3). The cost of production of a nursery piglet has followed a similar pattern than the nursery feed price across years (Fig. 3). On the other hand, pig company size was affecting most of the parameters studied, with the exception of nursery mortality rate, in different ways (Table 1). Thus, the lower the pig size company is, the better are average daily gain, feed conversion rate and drug and vaccine cost per nursery piglet. However, the opposite tendency is observed for nursery feed price where the highest value was observed for the smallest companies (Table 1). As a consequence, the cost per nursery piglet is similar for all the companies with the exception of companies between 5000 and 10000 sows whose value is higher than for the others. Finally, a complete descriptive statistics is shown by year in this phase (Additional file 1: Table S1 and S2).

### Fattening production phase

**Fig. 4:**
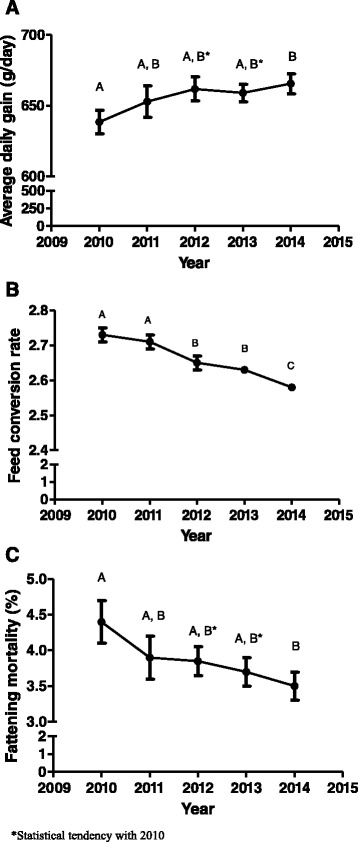
Temporal evolution of average daily gain (**a**), feed conversion rate (**b**) and fattening mortality (**c**) during fattening phase from 2010 to 2014 in Spain. Values with different superscripts differ significantly between years at *P* < 0.05

A steadily increase was observed in the average daily gain of fattening pigs from 2010 to 2014, with fattening mortality and fattening feed conversion rate steadily decreasing from 2010 to 2014 (Fig. 4). On the other hand, fattening feed price showed a close pattern than in the case of sow feed (Fig. 3). In this production phase, pig company size affected most of the parameters studied with the exception of feed conversion rate and mortality rate (Table 1). In general terms, the most widely used technical parameters (average daily gain and drug and vaccine cost during fattening phase) were observed for companies with less than 5000 sows. However, the bigger the pig size company is, the lower the feed price is. Globally, the total cost per pig is very similar across companies with the exception of 5000–10000 sow companies whose value is higher than for the other companies (Table 1). Finally, a complete descriptive statistics is provided by year in this phase (Additional file 1: Table S1 and S2).

### Total production phase

**Fig. 5:**
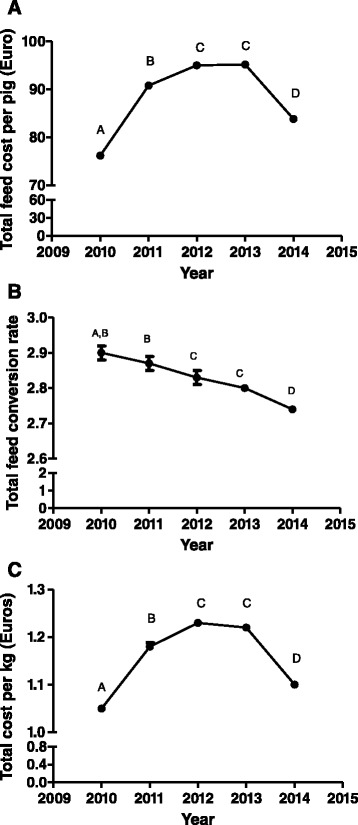
Temporal evolution of total feed cost per pig (**a**), feed conversion rate (**b**) and total cost per kilogram (**c**) during the whole rearing period from 2010 to 2014 in Spain. Values with different superscripts differ significantly between years at *P* < 0.05

Total feed conversion rate (the total feed use on a closed cycle farm divided by the total amount of kilograms of pigs produced) has been steadily decreasing from 2010 to 2014 (Fig. 5) whereas the total feed cost and the total cost per kilogram has significantly increased from 2010 to 2012 and decreased afterwards. On the other hand, pig company size was affecting most of the parameters studied, with the exception of total feed conversion rate in different ways (Table 1). Thus, the lower the pig size company is, the lower total drug and vaccine cost, total fixed cost and total reproduction cost per pig are. However, the opposite tendency is observed for the total feed cost per pig where the highest value was observed for the smallest companies. Total cost per produced Kg is very similar between companies with the exception of companies between 5000 and 10000 sows whose value is higher than for the rest of companies showing statistically significant differences with the biggest ones (Table 1). Finally, a complete descriptive statistics is shown by year during the whole rearing period (Additional file 1: Table S1 and S2).

### Linear model analysis

Using linear model analysis, number of piglets born alive (*p* < 0.0001), number of piglets weaned per sow (*p* < 0.0001) and number of piglets produced by sow and year (*P* < 0.0001) were positively associated with the year (the later the year is, the higher the value is) and there was also a significant interaction (*p* < 0.05) between the year and the pig company for them. On the other hand, fattening feed conversion rate (*p* = 0.003), total feed conversion rate (*p* = 0.006), fattening mortality (*p* = 0.049) and kilograms of sow feed per weaned piglet (*p* < 0.0001) were negatively associated with the year (the later the year is, the lower the value is). In this later case, a significant association was only observed between year and pig company for kilograms of sow feed per weaned piglet (*p* = 0.01).

For the rest of parameters, a significant association was not observed with the year.

## Discussion

### Production parameters

In the last five years in Spain, an increase in number of piglets born alive has been followed by a proportionate increase in number of piglets weaned by sow and number of piglets produced by sow and year (NPWY). This tendency has been also observed in other European countries during the same period but NPWY in Spain was always lower than in the main pig producing countries in Europe (Denmark, France, Germany and Netherlands) across the study period [8]. This steady increase in NPWY is probably reflecting an enormous success in the breeding program focus on increasing prolificacy during the last years without discarding the effect of other factors such as facilities and reproductive management improvement [9]. According to our results, NPWY will increase according to 0.5 pigs by year in Spain but we cannot foresee the duration of this increase in the long run. This increase will be higher than this average for some pig producing companies. This result suggests that the genetic improvement obtained could be different between pig selection companies. Thus, a higher improvement is expected in companies using hyperprolific breeds (Finestra A, personal communication). On the other hand, the best technical parameters in piglet production were obtained for companies with less than 5000 sows. A plausible explanation could be that better compliance with standard operation procedures can be applied in small farms with a low number of employees [10].

Performance indicators routinely used to measure pig productivity include average daily gain (ADG) and feed conversion rate (FCR) [11]. Both parameters in the last five years in Spain have globally improved during the fattening phase but not changed during the nursery phase. Moreover, we have observed variation in ADG and FCR between pig producing companies that could be attributed to management, health, genetic and facilities issues as described previously by other authors [12]. The same evolution for ADG and FCR has been observed for most of the main pig producing countries in Europe during the period 2010–2014 [8]. According to our results, total feed conversion rate will decrease 0.03 kg feed/kg gain per year in Spain. This improvement could be due to improvements in the diet, facilities, animal genetic and feeding management procedures. On the one hand, it was not observed any significant change in diet composition (energy and protein content per kg feed) across the study period and there is no data about general changes in facilities and feeding management that could affect to all the pig companies. However, the improvement of total feed conversion rate probably tallies with pig breeding programs focused on decreasing FCR as a selection goal and this factor is probably common for all the pig sector [9, 13]. In general terms, the best performance parameters were observed for companies with less than 5000 sows. Again, compliance with standard operation procedures can be better in small farms than in bigger ones with a high number of employees [10].

Mortality during the pig rearing period (nursery and fattening period) is directly associated with decreased profitability in swine operations [3]. This parameter has steadily decreased in Spain and Germany from 2010 to 2014. However, this parameter has scarcely changed in Netherlands, Denmark and France in the same period [8]. It is hard to explain this observation since the epidemiological situation is not different between European countries for the main respiratory pig diseases [14], likewise with the available tools (vaccines, hygiene procedures and antimicrobials availability) in order to develop medicine preventive programs to control them. Nevertheless, it cannot be overlooked that other factors such as the structure of the pig sector (farm size for example), the prevalence and/or virulence of other pig pathogens (e.g. digestive ones), the mortality level, and pig breeds could be critical factors in explaining observed differences between countries.

### Cost of production

The total cost of pig production can be divided into total feed cost (TFC), total fixed cost (TFIX), total reproduction cost (TREPC) and total drug and vaccine cost (DVCT) [15]. TFC resulted in approximately 67 % to 72 % of the total cost per pig in Spain, depending on the years as feed prices have suffered tremendous fluctuations over the last five years. Curiously, this percentage is always less than 64 % in other pig producing countries in Europe (Denmark, France, Germany and Netherlands), probably due to the fact that the feed price is always more expensive in Spain than in the other countries [8]. This difference could be explained by the chronic shortage of cereal production to provide enough raw materials for feed mills in Spain [16]. On the other hand, TFIX was cheaper in Spain (14–17 Euros/pig) than in the rest of European countries during the study period. Finally, TREPC and DVCT only resulted in between 1.6 and 4.2 % of the total cost per pig across years and countries. Globally, total cost per pig is cheaper in Spain than in the rest of European countries from 2012 to 2014. Thus, Spain is a very cost competitive country in the pig European production scenario. On the other hand, pig company size affected most of the pig production cost determinants. However, total cost per produced Kg is very similar between companies with the exception of companies between 5000 and 10.000 sows whose value is higher than for the rest of companies. Thus, it seems that a pig company with sow numbers between 5.000 and 10.000 does not have any advantage in terms of profitability in the future. Unfortunately, this result cannot be compared with other European countries because the pig company size is significantly higher in Spain than in other countries and for the different pig sector structure between European countries [8].

The cost of production of a weaned, nursery and fattening pig has followed a similar pattern as the sow, nursery and fattening feed price respectively in the last five years. Nevertheless, this cost would have continuously decreased if the feed price had been constant in the last five years due to the improvement observed for technical parameters in each production phase. Our results clearly demonstrate that feed conversion rate had the highest economic value in a high feed prices scenario as described by other authors [17].

## Conclusions

Pig production parameters have generally improved in the last five years. During the period 2010–2014, this improvement did not directly imply a reduction in pig production cost due to the high feed prices. Finally, pig company size is affecting not only production parameters but also pig production cost for companies with a sow numbers between 5.000 and 10.000.

## Methods

### Pig production companies included in this study

Between 61 and 107 pig production companies were included in this study from 2010 to 2014. These companies were operating in farrow-to-finish, two-site and three-site production systems and sizes varied from <1000 to >10.000 sows (Table 2). In any case, piglets born within the same week were managed as a cohort and they were weaned between three and four weeks of age. Then, they were moved to a nursery phase until they weighed an average of 19 kg (range: 16–23 kg); afterwards, they were allocated in finisher sites until reaching an average of 108 kg (range 102–116 kg), approximately. The pigs were then slaughtered in abattoir. The pig production companies were distributed across the country with a 75 % of the companies allocated in the biggest pig production area in Spain (Catalonia and Aragon). Moreover, the total number of sows included in this study varied from 334.000 to 546.000 sows during the period 2010 to 2014, respectively. These companies sent data on feed consumption, number of pig produced, expenses and census every month to a company specialized in analyzing these data from an economic perspective in several European countries (Sip consultors SL-- http://www.sipconsultors.com). Briefly, the collected data are described in Table 3 according to each pig production phase. Sip consultors SL standardized collected data and calculated cost and production parameters to obtain comparable values between the different pig production companies. Thus, the final weaning, nursery and fattening weight were standardized to 6, 19 and 108 kg for this research work, respectively. All the collected data each month were merged to obtain a yearly average value taking into account the pig production flow each month.

**Table 2:** Pig production company characteristics included in this research work from 2010 to 2014 in Spain

	Year				
	2010	2011	2012	2013	2014
Number of pig companies	61	57	72	87	107
Size company (sows)					
<1000	20	13	27	35	42
1000–5000	23	24	26	30	39
5000–10000	8	10	9	10	13
>10000	10	10	10	12	13
Total number of sows in database	334.307	349.695	353.503	460.413	546.868

**Table 3:** Monthly collected and calculated production parameters and pig production cost for each pig production company from 2010 to 2014 in Spain

Production phase	Parameter
Piglet production phase (gestation and lactation)	Number of piglets born alive (NBA)
	Preweaning mortality (%) (PM1)
	Number of piglets weaned by sow (NW)
	Number of piglets produced by sow and year (NPWY)
	Number of cycles by sow and year (NCS)
	Feed price for sows (Euros/tonne) (FP1)
	Kilograms of sow feed per weaned piglet (KFWP)
	Total kilograms of sow feed by year (TSF)
	Cost per Weaned piglet (Euros) (CWP1)
Nursery phase (from weaning to 19 kg of bw)	Nursery average daily gain (g/day) (ADG2)
	Nursery feed conversion rate (FCR2)
	Nursery mortality (%) (NM2)
	Feed price for nursery (Euros/tonne) (FP2)
	Cost per nursery piglet (Euros) (CNP2)
	Drug and vaccine cost per nursery piglet^a^ (DVCNP2)
Fattening phase (from 19 to 108 kg of bw)	Fattening average daily gain (g/day) (ADG3)
	Fattening feed conversion rate (FCR3)
	Fattening mortality (%) (FM3)
	Feed price for fattening (Euros/tonne) (FP3)
	Total cost per pig (Euros) (TCP3)
	Drug and vaccine cost during fattening phase (DVCFP3)
Whole production phase	Total feed conversion rate (FCRT)
	Total cost per produced Kg (TCK)
	Total feed cost (Euros) per pig (TFC)
	Total drug and vaccine cost (Euros) per pig (DVCT)
	Total fixed cost (Euros) per pig (TFIXC)
	Total reproduction cost (Euros) per pig (TREPC)

^a^Cost in piglet production phase of drug and vaccines included

### Statistical analyses

All statistical analyses were carried out using the SAS system V.9.1.3 (SAS institute Inc, Cary, NC, USA). A statistics descriptive (mean, standard error of the mean (SEM), variation coefficient and 95 % confidence interval) was calculated for each parameter during the period 2010 to 2014. Each pig company was used as the experimental unit for further analysis. The significance level (*p*) was set at 0.05 with statistical tendencies reported when *P* < 0.10. Shapiro Wilk’s and Levene tests were used to evaluate the normality of the distribution of the variables and the homogeneity of variances, respectively. An ANOVA (with Student’s T-test to compare each pair of values) or Wilcoxon test (with two-pair comparisons) was used to analyse the association between continuous normally or non-normally distributed variables and year and pig company size, respectively. Finally, a linear model was performed to evaluate the association between the production parameters and the year taking into account that the data was recorded each year (repeated measures) and the potential interaction between year and pig company. For this analysis, it was only included only the pig companies with data during the whole study period.

## Additional file

Additional file 1:
**Table S1.** Mean, SEM, variation coefficient and 95 % confidence interval of production parameters and pig production cost from 2010–2012 in Spain. Abbreviations are defined in Table 3. **Table S2**. Mean, SEM, variation coefficient and 95 % confidence interval of production parameters and pig production cost from 2013–2014 in Spain. Abbreviations are defined in Table 3. **Figure S1.** Temporal evolution of average daily gain (A), feed conversion rate (B) and mortality (C) during nursery production phase from 2010 to 2014 in Spain. Values with different superscripts differ significantly between years at *P* < 0.05. (DOCX 45 kb)

## References

[1] Faostat 2013. http://faostat3.fao.org/home/E. Accessed 11^th^ September 2014.

[2] De Lange FM, Dewey C, Straw BE, Zimmerman J, D’Allaire S, Taylor DJ (2009). Management of growing-finishing pigs, chapter 6, Disease of swine.

[3] Holden P (1991). Swine costs and production. Agri-practice.

[4] Losinger WC (2005). Economic impacts of reduced pork production associated with the diagnosis of Actinobacillus pleuropneumoniae on grower/finisher swine operations in the United States. Prev Vet Med.

[5] Fenwick B (2004). Relationship between vaccination and management in assuring profitable pork production. Animal Health Res Rev.

[6] Alarcon P, Rushton J, Wieland B (2013). Cost of post-weaning multi-systemic wasting syndrome and porcine circovirus type-2 subclinical infection in England - an economic disease model. Prev Vet Med.

[7] Alarcon P, Rushton J, Nathues H, Wieland B (2013). Economic efficiency analysis of different strategies to control post-weaning multi-systemic wasting syndrome and porcine circovirus type 2 subclinical infection in 3-weekly batch system farms. Prev Vet Med.

[8] Interpig report 2014. http://www.sipconsultors.com/en/home. Accessed 19th September 2014.

[9] Danbreed report. http://www.danbredinternational.dk/breeding-goal. Accessed 20^th^ September 2014.

[10] Bonneau M, de Greef K, Brinkman D, Cinar MU, Dourmad JY, Edge HL (2014). Evaluation of the sustainability of contrasted pig farming systems: the procedure, the evaluated systems and the evaluation tools. Animal.

[11] Black JL, Giles LR, Wynn, PC, Knowles AG, Kerr CA, Jones MR, Strom AD, Gallagher NL, Eamens GJ. Factors limiting the performance of growing pigs in commercial environments, in: Proceedings of the Eighth Biennial Conference of the Australasian Pig Science Association (APSA), November, 2001, Adelaide Werribee, Victoria, pp. 150–170.

[12] Magowan E, McCann ME, Beattie VE, McCracken KJ, Henry W, Smyth S (2007). Investigation of growth rate variation between commercial pig herds. Animal.

[13] PIC report 2014. http://www.picgenus.com/home.aspx. Accessed on 12^th^ September 2014.

[14] Fraile L, Alegre A, López-Jiménez R, Nofrarías M, Segalés J (2010). Risk factors associated with pleuritis and cranio-ventral pulmonary consolidation in slaughter-aged pigs. Vet J.

[15] Ilari-Antoine E, Bonneau M, Klauke TN, Gonzàlez J, Dourmad JY, De Greef K (2014). Evaluation of the sustainability of contrasted pig farming systems: economy. Animal..

[16] Spanish ministry of Agriculture report 2014. http://www.magrama.gob.es/es/agricultura/temas/producciones-agricolas/cultivos-herbaceos/cereales/. Accessed on 18^th^ September 2014.

[17] Dube B, Mulugeta SD, Dzama K (2013). Integrating economic parameters into genetic selection for Large White pigs. Animal..

