# Supplementary material for: Production parameters and pig production cost: temporal evolution 2010–2014
Source: Porcine Health Manag. 2016 Apr 11;2:11. doi: 10.1186/s40813-016-0027-0 (PMC5382395; doi:10.1186/s40813-016-0027-0)
Supplement: Additional file 1: — Table S1. Mean, SEM, variation coefficient and 95 % confidence interval of production parameters and pig production cost from 2010–2012 in Spain. Abbreviations are defined in Table 3. Table S2. Mean, SEM, variation coefficient and 95 % confidence interval of production parameters and pig production cost from 2013–2014 in Spain. Abbreviations are defined in Table 3. Figure S1. Temporal evolution of average daily gain (A), feed conversion rate (B) and mortality (C) during nursery production phase from 2010 to 2014 in Spain. Values with different superscripts differ significantly between years at P < 0.05. (DOCX 45 kb) [file 40813_2016_27_MOESM1_ESM.docx]

| **Additional file Table S1**. Mean, SEM, variation coefficient and 95% confidence interval of production parameters and pig production cost from 2010-2012 in Spain. Abbreviations are defined in Table 2. | | | | | | | | | | | | |
| --- | --- | --- | --- | --- | --- | --- | --- | --- | --- | --- | --- | --- |
|  | Year | | | | | | | | | | | |
|  | 2010 | | | | 2011 | | | | 2012 | | | |
| Variable | Mean | SEM | Variation coefficient | Confidence Interval | Mean | SEM | Variation coefficient | Confidence Interval | Mean | SEM | Variation coefficient | Confidence Interval |
| FP1 | 214.15 | 1.87 | 6.80 | (210.42-217.88) | 264.96 | 1.97 | 5.61 | (261.01-268.90) | 283.49 | 1.55 | 4.64 | (280.40-286.58) |
| TSF | 1124.70 | 11.25 | 7.75 | (1147.22-1102.18) | 1132.55 | 8.70 | 5.75 | (1115.11-1150.01) | 1133.91 | 7.47 | 5.59 | (1119.01-1148.82) |
| KFWP | 43.17 | 0.66 | 12.30 | (45.76-48.51) | 45.99 | 0.61 | 9.94 | (44.78-47.20) | 45.94 | 0.43 | 7.96 | (45.08-46.81) |
| NBA | 11.68 | 0.10 | 6.51 | (11.48-11.87) | 11.84 | 0.08 | 5.57 | (11.66-12.01) | 11.99 | 0.08 | 6.01 | (11.82-12.17) |
| MP1 | 0.12 | 0.01 | 21.67 | (0.11-0.13) | 0.11 | 0.01 | 22.62 | (0.11-0.12) | 0.12 | 0.01 | 24.62 | (0.11-0.13) |
| NCY | 2.33 | 0.01 | 4.55 | (2.30-2.36) | 2.34 | 0.01 | 3.85 | (2.32-2.37) | 2.34 | 0.01 | 3.85 | (2.31-2.36) |
| NW | 10.25 | 0.08 | 5.76 | (10.11-10.50) | 10.48 | 0.07 | 5.06 | (10.33-10.62) | 10.56 | 0.07 | 5.40 | (10.42-10.69) |
| NWPY | 23.96 | 0.26 | 8.51 | (23.43-24.48) | 24.64 | 0.24 | 7.47 | (24.15-25.12) | 24.69 | 0.22 | 7.53 | (24.25-25.12) |
| CWP1 | 25.96 | 0.36 | 10.95 | (25.23-26.69) | 27.73 | 0.34 | 9.19 | (27.04-28.41) | 28.45 | 0.29 | 8.73 | (27.87-29.04) |
| FP2 | 415.33 | 4.86 | 9.06 | (405.62-425.05) | 457.42 | 5.74 | 9.39 | (445.92-468.92) | 479.17 | 5.12 | 9.00 | (468.96-489.38) |
| FCR2 | 1.67 | 0.02 | 7.50 | (1.64-1.70) | 1.67 | 0.02 | 6.77 | (1.64-1.70) | 1.67 | 0.01 | 6.66 | (1.64-1.69) |
| ADG2 | 293.01 | 4.62 | 11.71 | (283.74-302.29) | 291.62 | 4.59 | 11.35 | (282.40-300.84) | 284.75 | 4.91 | 13.82 | (274.91-294.58) |
| NM2 | 0.03 | 0.01 | 34.28 | (0.029-0.035) | 0.03 | 0.01 | 42.24 | (0.027-0.034) | 0.03 | 0.01 | 48.02 | (0.030-0.037) |
| DVCNP2 | 3.17 | 0.12 | 28.85 | (2.93-3.41) | 3.24 | 0.11 | 25.63 | (3.02-3.46) | 3.27 | 0.11 | 27.64 | (3.06-3.49) |
| CNP2 | 39.62 | 0.47 | 9.21 | (38.67-40.56) | 42.44 | 0.45 | 7.86 | (41.55-43.34) | 43.38 | 0.36 | 7.09 | (42.65-44.11) |
| FP3 | 233.70 | 1.62 | 4.59 | (230.44-236.97) | 286.60 | 1.71 | 4.01 | (283.15-290.04) | 306.80 | 1.71 | 4.27 | (303.78-310.21) |
| FCR3 | 2.73 | 0.02 | 5.06 | (2.69-2.77) | 2.71 | 0.02 | 5.02 | (2.67-2.75) | 2.65 | 0.02 | 4.34 | (2.62-2.68) |
| ADG3 | 638.45 | 8.46 | 7.38 | (621.16-655.73) | 652.92 | 11.01 | 8.77 | (630.28-675.56) | 661.75 | 8.55 | 7.86 | (644.40-679.11) |
| NM3 | 0.04 | 0.01 | 35.82 | (0.04-0.05) | 0.04 | 0.01 | 34.06 | (0.033-0.041) | 0.04 | 0.01 | 41.78 | (0.034-0.043) |
| DVCNP3 | 1.57 | 0.09 | 37.61 | (1.38-1.75) | 1.68 | 0.12 | 48.66 | (1.43-1.92) | 1.58 | 0.11 | 51.14 | (1.37-1.79) |
| CNP3 | 113.33 | 0.86 | 5.11 | (111.59-115.07) | 127.26 | 0.88 | 4.64 | (125.49-129.03) | 130.78 | 0.67 | 3.95 | (129.43-132.13) |
| TCK | 1.05 | 0.01 | 5.15 | (1.03-1.06) | 1.18 | 0.01 | 4.56 | (1.17-1.20) | 1.23 | 0.01 | 4.00 | (1.21-1.24) |
| FCRT | 2.90 | 0.02 | 4.86 | (2.86-2.95) | 2.87 | 0.02 | 4.63 | (2.83-2.91) | 2.83 | 0.02 | 4.10 | (2.80-2.86) |
| TFC | 76.18 | 0.68 | 5.97 | (74.81-77.54) | 90.82 | 0.74 | 5.47 | (89.32-92.31) | 94.97 | 0.67 | 5.41 | (93.63-96.31) |
| DVCT | 5.04 | 0.22 | 29.86 | (4.59-5.95) | 4.97 | 0.21 | 28.96 | (4.53-5.40) | 4.86 | 0.20 | 31.35 | (4.47-5.26) |
| TFIXC | 29.27 | 0.55 | 12.50 | (28.17-30.37) | 28.49 | 0.45 | 10.57 | (27.57-29.40) | 28.56 | 0.36 | 9.67 | (27.84-29.28) |
| TREPC | 2.76 | 0.17 | 41.05 | (2.42-3.10) | 2.67 | 0.13 | 33.61 | (2.40-2.94) | 2.38 | 0.12 | 36.78 | (2.15-2.61) |

| **Additional file Table S2**. Mean, SEM, variation coefficient and 95% confidence interval of production parameters and pig production cost from 2013-2014 in Spain. Abbreviations are defined in Table 2. | | | | | | | | |
| --- | --- | --- | --- | --- | --- | --- | --- | --- |
|  | Year | | | | | | | |
|  | 2013 | | | | 2014 | | | |
| Variable | Mean | SEM | Variation coefficient | Confidence Interval | Mean | SEM | Variation coefficient | Confidence Interval |
| FP1 | 280.29 | 1.57 | 5.23 | (277.17-283.41) | 246.97 | 1.39 | 5.81 | (244.22-249.72) |
| TSF | 1143.75 | 7.63 | 6.22 | (1128.57-1158.92) | 1132.98 | 7.40 | 6.75 | (1118.32-1147.65) |
| KFWP | 45.48 | 0.44 | 9.01 | (44.61-46.36) | 43.98 | 0.39 | 9.11 | (43.22-44.75) |
| NBA | 12.33 | 0.09 | 7.14 | (12.14-12.52) | 12.55 | 0.10 | 7.86 | (12.36-12.74) |
| MP1 | 0.119 | 0.01 | 27.97 | (0.112-0.127) | 0.12 | 0.01 | 27.98 | (0.113-0.125) |
| NCY | 2.33 | 0.01 | 3.86 | (2.31-2.35) | 2.35 | 0.01 | 3.32 | (2.33-2.36) |
| NW | 10.83 | 0.07 | 6.09 | (10.70-10.98) | 11.04 | 0.07 | 6.92 | (10.89-11.18) |
| NWPY | 25.26 | 0.21 | 7.60 | (24.86-25.68) | 25.88 | 0.20 | 7.84 | (25.49-26.27) |
| CWP1 | 28.56 | 0.30 | 9.72 | (27.97-29.15) | 26.24 | 0.25 | 9.94 | (25.74-26.74) |
| FP2 | 487.98 | 5.25 | 9.81 | (477.34-498.25) | 452.98 | 4.30 | 9.39 | (444.45-461.50) |
| FCR2 | 1.67 | 0.02 | 8.39 | (1.64-1.70) | 1.65 | 0.01 | 7.86 | (1.63-1.68) |
| ADG2 | 289.29 | 4.76 | 14.25 | (279.80-298.77) | 291.95 | 3.55 | 12.03 | (284.90-299.00) |
| NM2 | 0.032 | 0.01 | 46.58 | (0.029-0.036) | 0.031 | 0.01 | 47.25 | (0.028-0.033) |
| DVCNP2 | 3.47 | 0.10 | 27.22 | (3.26-3.68) | 3.339 | 0.10 | 28.33 | (3.15-3.53) |
| CNP2 | 43.95 | 0.37 | 7.55 | (43.22-44.68) | 41.024 | 0.33 | 7.86 | (40.38-41.67) |
| FP3 | 308.22 | 1.58 | 4.25 | (305.07-311-36) | 277.308 | 1.27 | 4.08 | (274.78-279.84) |
| FCR3 | 2.66 | 0.01 | 3.68 | (2.61-2.66) | 2.583 | 0.01 | 4.14 | (2.56-2.61) |
| ADG3 | 659.02 | 6.18 | 6.22 | (646.55-671.49) | 668.919 | 6.33 | 6.83 | (656.21-681.63) |
| NM3 | 0.037 | 0.01 | 35.33 | (0.034-0.040) | 0.034 | 0.01 | 35.47 | (0.032-0.037) |
| DVCNP3 | 1.55 | 0.11 | 57.96 | (1.34-1.77) | 1.522 | 0.09 | 54.80 | (1.33-1.71) |
| CNP3 | 131.54 | 0.75 | 4.79 | (130.04-133.04) | 117.73 | 0.61 | 4.58 | (116.53-118.94) |
| TCK | 1.22 | 0.01 | 4.74 | (1.21-1.24) | 1.10 | 0.01 | 4.55 | (1.09-1.11) |
| FCRT | 2.80 | 0.01 | 3.89 | (2.78-2.83) | 2.74 | 0.01 | 4.01 | (2.72-2.77) |
| TFC | 95.22 | 0.53 | 4.62 | (94.16-96.29) | 83.88 | 0.50 | 5.28 | (82.89-84.87) |
| DVCT | 5.043 | 0.19 | 31.93 | (4.66-5.43) | 4.980 | 0.18 | 31.18 | (4.63-5.33) |
| TFIXC | 28.33 | 0.38 | 11.04 | (27.58-29.08) | 27.44 | 0.33 | 10.51 | (26.79-28.09) |
| TREPC | 2.65 | 0.13 | 39.55 | (2.39-2.90) | 2.65 | 0.10 | 34.69 | (2.44-2.58) |

**Additional file Figure** **S1** Temporal evolution of average daily gain (A), feed conversion rate (B) and mortality (C) during nursery production phase from 2010 to 2014 in Spain. Values with different superscripts differ significantly between years at *P*<0.05.

A

B

C
